# OmicIntegrator: A Simple and Versatile Tool for Meta-Analysis

**DOI:** 10.3390/plants15020334

**Published:** 2026-01-22

**Authors:** Iván Federico Berco Gitman, Cecilia Eugenia María Grossi, Denise Soledad Arico, María Agustina Mazzella, Rita María Ulloa

**Affiliations:** 1 Instituto de Investigaciones en Ingeniería Genética y Biología Molecular “Dr. Héctor Torres” (INGEBI), Consejo Nacional de Investigaciones Científicas y Técnicas (CONICET), Vuelta de Obligado 2490, 2do piso, Ciudad Autónoma de Buenos Aires 1428, Argentina; igitman@ingebi-conicet.gov.ar (I.F.B.G.); cgrossi@ingebi-conicet.gov.ar (C.E.M.G.); denise.s.arico@gmail.com (D.S.A.); 2 Laboratoire de Recherche en Sciences Végétales, Université de Toulouse, CNRS, UPS, 31320 Auzeville Tolosane, France; 3 Laboratoire Reproduction et Développement des Plantes, ENS de Lyon, CNRS, INRAE, UCBL, 69364 Lyon, France; 4 Departamento de Química Biológica, Universidad de Buenos Aires (UBA), Ciudad Autónoma de Buenos Aires 1428, Argentina

**Keywords:** *Arabidopsis thaliana*, protein kinases, protein phosphatases, skotomorphogenesis, transcriptomic, proteomic and phosphoproteomic integrative meta-analysis

## Abstract

We developed OmicIntegrator, a broadly adaptable pipeline designed to standardize and integrate publicly available transcriptomic, proteomic, and phosphoproteomic datasets. We applied this workflow to *Arabidopsis thaliana* etiolated seedlings to identify protein kinases and phosphatases relevant to skotomorphogenic development, a phase during which seedlings rely on tightly regulated signaling networks to ensure survival in darkness. This meta-analysis provided a comprehensive view of gene and protein expression, revealing discrepancies between transcript and protein abundance, suggesting post-transcriptional and post-translational regulation. By integrating multiple datasets, OmicIntegrator reduces experimental bias and enables the detection of phosphorylation events that may be missed in single-condition studies. Distinct phosphorylation patterns were detected across different protein kinase families. Motif enrichment analysis showed a strong overrepresentation of RxxS motifs among phosphosites in protein phosphatases and microtubule-associated proteins, consistent with potential regulation by calcium-dependent protein kinases (CPKs). Across omics layers, CPK3 and CPK9 repeatedly emerged as prominent candidates, highlighting them as priorities for future functional studies in skotomorphogenesis. Overall, our results demonstrate the power of OmicIntegrator as a flexible framework to contextualize signaling landscapes and identify robust patterns and candidate genes and for generating testable hypotheses from integrated multi-omics data in plant developmental biology.

## 1. Introduction

In recent years, numerous studies and reviews have highlighted the potential of multi-omics integration to capture regulatory complexity beyond individual molecular layers [1,2,3,4,5]. In parallel, a growing number of computational frameworks and platforms, including Bigomics Analytics, mixOmics, and CORDIS, have developed tools or provide services to facilitate the integrated analysis of high-dimensional datasets, particularly in the field of biomedicine. In plant research, integrated analyses of matched multi-omic datasets generated under uniform experimental conditions and produced within the same laboratory or research consortium have been performed in *Arabidopsis thaliana* [6], rice [7,8], maize [9] and poplar [10], among others.

An important advantage of integrating omics datasets generated by independent laboratories is the possibility of drawing robust biological conclusions under a double-blind framework, where data producers and data analysts remain unaware of each other’s hypotheses and expectations. Despite the growing availability of public databases and large-scale datasets, the main limitation in omics research now lies less in data production, which has become increasingly accessible and scalable, and more in the systematic, unbiased analysis and integration of these massive datasets to extract meaningful biological insights. Multiple strategies have been developed to integrate multi-omics data in plant research, including sequential, pathway-based, network-based, machine learning, and Bayesian approaches. However, data heterogeneity and lack of standardization are among the major challenges in the analysis of multi-omic data [11]. Thus, it is important to complement these existing strategies by providing a standardized and transparent framework for harmonizing transcriptomic, proteomic, and phosphoproteomic datasets generated by different laboratories and platforms.

*Arabidopsis thaliana* is a predominant model system for studying plant molecular, cellular, and evolutionary biology. Its widespread use has led to the development of multiple public databases, software tools, and online platforms freely available to the plant community [12]. Skotomorphogenesis is an early developmental phase that demands energy consumption and is critical for the establishment of seedlings beneath the soil. After reaching the surface, light induces photomorphogenesis mainly through phytochrome and cryptochrome photoreceptors, enabling seedlings to become autotrophic [13,14,15,16]. Reversible protein phosphorylation and calcium (Ca^2+^) play a key role in the response of plants to environmental and endogenous stimuli [17,18,19,20]. This dynamic post-translational modification can modulate protein conformation, activation state, turnover, subcellular localization, and interactions. It involves protein kinase (PK) activities, which transfer the gamma phosphate from ATP to specific serine (S), threonine (T), or tyrosine (Y) residues, and protein phosphatase (PP) activities responsible for the removal of phosphate groups [21]. The Arabidopsis genome is predicted to encode roughly 1000 PKs and 150 PPs [22,23]. CPKs are the main Ca^2+^ sensor-transducers in plants; these proteins possess EF-hand calcium-binding motifs and an S/T PK domain, enabling them to combine sensing and responding activities. This multigenic family, first described in *Arabidopsis thaliana*, comprises 34 members [24] that exhibit substantial similarity except for the N-terminal and C-terminal (NT and CT) variable domains. The NT is critical for subcellular localization [24,25] and is important for substrate recognition [26]. Evidence for CPK function during early seedling development remains sparse, spanning early studies in *Pharbitis nil* and more recent reports in Arabidopsis [27,28]. A family-wide multi-omic analysis can, therefore, provide deeper insight into CPK roles in etiolated seedlings.

Here, we present OmicIntegrator, a standardized framework for integrating heterogeneous public multi-omic datasets, applied to etiolated Arabidopsis seedlings using PKs and PPs as an illustrative module to uncover promising candidates for future functional studies during this developmental stage. By implementing explicit normalization procedures, confidence thresholds, and adjustable filtering criteria, this pipeline enables robust cross-dataset comparisons and supports the generation of high-confidence, reusable multi-omic resources suitable for downstream functional analyses.

## 2. Results

### 2.1. Integration of Multi-Omic Datasets and Threshold Settings

**Figure 1:**
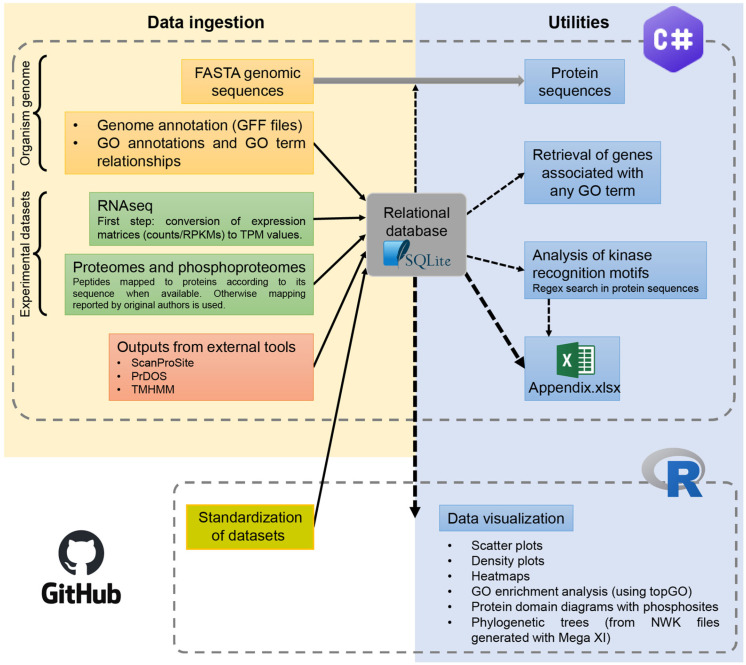
OmicIntegrator workflow diagram. Data ingestion into a relational SQLite database is handled in C#. After loading the genome of the organism of interest (GFF files, chromosomal FASTA sequences and GO annotations, orange boxes), omics datasets corresponding to different experimental conditions can be imported (green boxes). Dataset standardization for each condition is carried out in the R environment, and the normalized data are loaded back into the database (yellow box). Results of external analytical tools (e.g., ScanProSite, TMHMM-2.0, and NORSp) can also be integrated (pink box). The C# program provides multiple data processing and export utilities (blue boxes) and can generate outputs in Excel format. Data visualization is performed in R, using either these outputs or direct queries to the SQLite database. All source code, scripts, and datasets used in this study are available on GitHub (https://github.com/ifgitman/OmicIntegrator, accessed on 20 October 2025).

Omic datasets generated by different laboratories are often published in highly heterogeneous formats. To address this, we developed a relational database schema that accommodates raw data and enables downstream analyses through unified algorithms. Only minimal code is needed to adapt different data source formats to the database schema, enabling the usage of this framework for the study of different experimental conditions. The overall workflow is described in Figure 1. We selected publicly available RNA-seq, proteomic, and phosphoproteomic datasets from *Arabidopsis thaliana* etiolated seedlings and used OmicIntegrator to manage these data layers and extract meaningful information related to reversible phosphorylation-mediated signaling.

**Figure 2:**
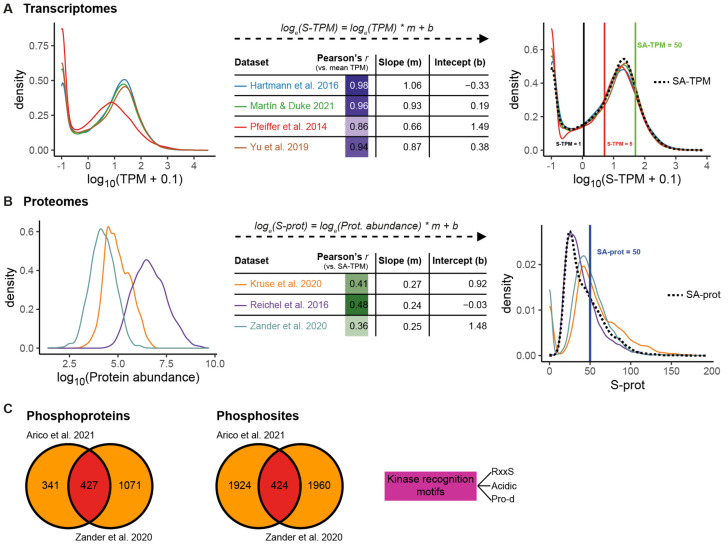
Standardization of omic datasets from *Arabidopsis thaliana* dark-grown seedlings. (**A**) **Left:** Distribution of TPM values for 27,533 genes across four RNA-seq datasets [29,30,31,32]. **Middle:** Pearson correlation coefficients and linear parameters (m, b) derived from linear regressions (in natural logarithmic scale) of each dataset against the mean TPM across datasets. **Right:** Distribution of standardized TPM values (S-TPM), calculated as *log_e_*(*S-TPM*) = *log_e_*(*TPM*) * *m* + *b*. The standardized average TPM (SA-TPM; dotted line) represents the mean S-TPM across datasets. The red line indicates the threshold for confident transcript detection (S-TPM > 5 in at least three experiments). The black line indicates transcripts below the detection threshold (S-TPM values < 1 in at least three experiments). The green line indicates the threshold for highly abundant transcripts (SA-TPM > 50). (**B**) **Left:** Distribution of protein abundance across three datasets [33,34,35]. **Middle:** Pearson correlation coefficients and linear parameters (m, b) derived from linear regressions (in natural logarithmic scale) of the protein abundance values in each experiment and the corresponding SA-TPM for genes with detectable transcript levels. **Right:** Distribution of standardized protein abundance values (S-prot) calculated as *log_e_*(*S-prot*) = *log_e_*(*Prot. abundance*) * *m* + *b*. S-prot values were averaged to compute a standardized average protein abundance (SA-prot) for each gene. The dotted line corresponds to the distribution of SA-prot values for proteins that meet the protein-detection criteria. The blue line indicates the threshold for highly abundant proteins (SA-prot > 50). (**C**) Venn diagram of phosphoproteins (left) and phosphosites (right) detected in both phosphoproteomes [34,36]. Analysis of kinase recognition motifs for the identified phosphosites was performed [37].

The distribution of the TPM values from the four RNA-seqs prior to standardization is shown in Figure 2A, left panel. Following normalization, the distributions of the S-TPM (standardized TPM, see M&M) values largely overlapped, demonstrating the high reproducibility of the transcriptomic data. The transcriptome from Pfeiffer et al. (2014) [29] showed a weaker correlation with the other datasets (Figure S1 and Figure 2A middle panel). Its S-TPM distribution reveals a deficit of very low-abundant transcripts (S-TPM < 1), reflecting reduced sensitivity (Figure 2A, right panel). We applied stringent cut-offs to maximize confidence in transcript detection. A gene was considered transcriptionally detected if it showed S-TPM values > 5 in at least three RNA-seq datasets, and below the detection thresholds if S-TPM values were < 1 in at least three experiments. Genes with S-TPM values between 1 and 5 fall into an ambiguous transcriptional range, in which transcriptional activity cannot be confidently assigned based on the available datasets. Values within this range may reflect low-level basal transcription or condition-specific expression that is not consistently detected across experiments. Genes with SA-TPM > 50 (standardized average TPM, see M&M) were classified as abundantly transcribed (Figure 2A, right panel).

Both the distribution of protein abundance for each proteome and the linear regressions are shown in Figure 2B (left and middle panels) and Figure S2. After standardization, the overlap between proteomic datasets was lower than that observed for the RNA-seq datasets (Figure 2B). The proteome from Reichel et al. (2016) [33] displays a wider dynamic range and higher sensitivity, yielding a stronger correlation with the transcriptomic data (Figure 2B, middle panel). It detected proteins that were not identified in the other two proteomes, and consequently, in the right panel of Figure 2B, the distribution of S-prot values is shifted toward the left. As in transcript analysis, we applied a stringent cut-off here as well, at the risk of excluding some true positives. Protein presence was defined based on conservative detection criteria, requiring identification in at least two independent proteome datasets (S-prot > 0) with unequivocal assignment of exclusive peptides or phosphopeptides. Proteins were considered not detected when S-prot was 0 across all three proteomes. Proteins that did not meet these criteria were classified as uncertain, reflecting low-confidence detection. Proteins with SA-prot > 50 were classified as highly abundant (Figure 2B, right panel).

Regarding the phosphoproteomic datasets, intersection of the two studies revealed a limited overlap, comprising approximately 25% of the identified phosphoproteins and ~10% of the detected phosphosites (Figure 2C), consistent with the highly dynamic and context-dependent nature of reversible phosphorylation. This overlapping fraction was defined as a high-confidence phosphoproteome; however, to capture a broader view of phosphorylation events, we included all phosphoproteins and phosphosites detected in either dataset for downstream analyses, provided that the corresponding proteins met our detection criteria. Importantly, the identified phosphosites represent potential regulatory events present at this developmental stage, rather than a complete or temporally resolved phosphorylation map. While dynamic and time-resolved phosphoproteomic analyses will be required to define signaling order and kinetics, the integrated dataset presented here provides a robust foundation for prioritizing candidates and for future functional and temporal studies. To infer potential upstream kinases, we analyzed conserved phosphosite consensus sequences, as large-scale studies in plants have identified characteristic kinase recognition motifs [37]. The complete genome-wide integration output is provided in File S1.

### 2.2. Multi-Omic Datasets Reveal Widespread Post-Transcriptional Regulation in Etiolated Seedlings

**Figure 3:**
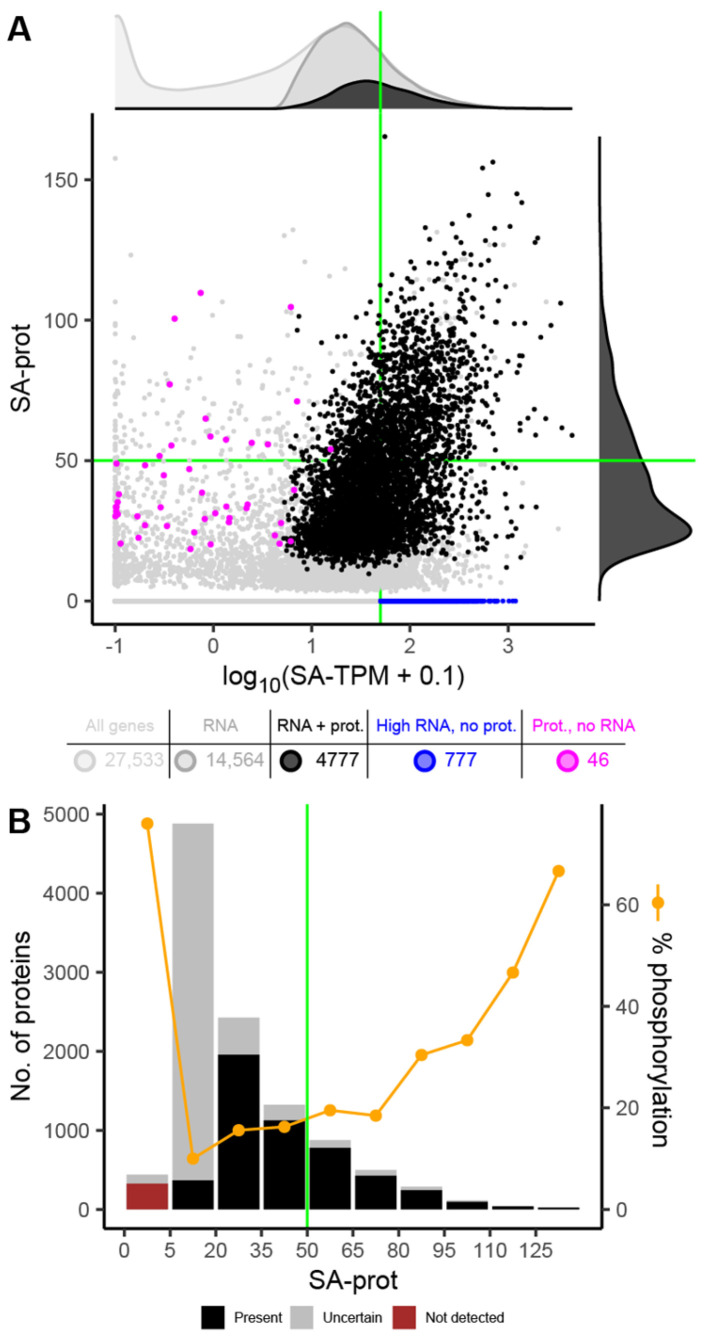
Multi-Omic Integration of Transcript, Protein, and Phosphoprotein Profiles in Dark-Grown Seedlings. (**A**) Scatter plot of transcript versus protein abundance for 27,533 protein-coding genes. Green lines mark the thresholds for high transcript or high protein abundance. (⏺) Proteins with corresponding transcripts; (⏺) high transcript levels without detected protein; (⏺) proteins without detected transcripts (stored proteins); (⏺) below thresholds or with ambiguous peptides. Gene counts per category are shown in the table below, and their distributions are summarized in kernel density plots at the margins: top, all genes (light gray), transcribed genes (dark gray), and transcripts with corresponding protein (black); right: proteins with transcripts. (**B**) Protein distribution according to SA-prot values and percentage of phosphorylated proteins for each category. Black bars: highly confident proteins (detected in at least two datasets, with exclusive peptides); gray bars: uncertain proteins (detected in only one dataset and/or without exclusive peptides); brown bar: not detected proteins (SA-prot = 0). The green line marks the threshold for high protein abundance.

Analysis of the selected RNA-seq datasets showed that 14,564 genes of the 27,533 protein-coding genes (53%) were transcriptionally active in etiolated seedlings, with 3692 exhibiting high transcript levels (SA-TPM > 50; dots to the right of the green line in Figure 3A, Table 1). However, only one-third of these transcribed genes (4777 out of 14,564) had corresponding proteins (Figure 3A, Table 1). Notably, 21% of the highly transcribed genes (777 out of 3692) were not detected in the proteomes (blue dots in Figure 3A), reflecting a combination of biological and technical factors, including post-transcriptional regulation, rapid protein turnover, developmental timing, and the detection limits inherent to large-scale proteomic analyses. GO analysis of genes with SA-TPM > 100 but no protein detection (SA-prot = 0) revealed significant enrichment for terms related to photosynthesis and chloroplast thylakoid membranes (Table S1). Conversely, among the 5043 proteins confidently detected across the three proteomic datasets, 46 corresponded to transcripts with S-TPM < 1 in at least three experiments (magenta dots in Figure 3A, Table 1). These “stored proteins” were enriched for GO terms associated with post-embryonic development, carbohydrate metabolism, and nutrient reservoir activity (Table S2). In agreement, eFP Browser data indicate that 20 of these genes are predominantly expressed in dry or imbibed seeds (Figure S3). While these discrepancies may partly reflect technical limitations inherent to large-scale proteomic analyses, their recurrent occurrence across specific gene families and functional categories is consistent with biologically regulated post-transcriptional control, potentially involving selective translation, rapid protein turnover, or developmental timing effects.

Most reliably detected proteins (black bars in Figure 3B) had SA-prot values between 20 and 50, while 1596 were classified as highly abundant (SA-prot > 50). By contrast, most uncertain proteins had SA-prot values between 5 and 20 (gray bars in Figure 3B). We identified 1839 phosphoproteins in at least one of the two available phosphoproteomic datasets from dark-grown seedlings [34,36], with 427 consistently detected in both (Figure 2C). Strikingly, 325 phosphoproteins were not detected in any proteome (brown bar in Figure 3B), including 37 with high transcript levels (Table S3). It is tempting to suggest that phosphorylation could precede protein degradation, but it also may reflect rapid protein turnover, transient expression, or technical factors related to peptide enrichment and detection sensitivity. After filtering according to protein detection criteria, 1019 phosphoproteins (with 2827 phosphosites) were classified as bona fide (Table 1). At this developmental stage, phosphoproteins accounted for 20.2% of the proteome, a percentage that increased significantly (χ^2^ = 52.8, *p* < 0.00001) among proteins with SA-prot > 80 (Figure 3B). Analysis of the phosphoproteome revealed that 23.1% of the phosphosites matched consensus motifs for GSK3, CDK, and/or MAPK activities; 13.5% for Ca^2+^/CaM-PKs and CPKs; and 19.6% for CK2 (Table 1). These assignments are based on shared consensus motifs that can be targeted by multiple kinases within a family or subfamily and, thus, reflect combined rather than individual kinase activities.

By standardizing multi-omic datasets from etiolated seedlings, we observed that many highly expressed genes lacked detected protein products, while others accumulated at the protein levels in the absence of corresponding transcripts. Together, these discrepancies between transcript and protein levels are consistent with widespread post-transcriptional and post-translational regulation operating during skotomorphogenesis.

### 2.3. Selective Expression of Protein Kinases in Etiolated Seedlings

The Arabidopsis genome encodes 1055 genes annotated with protein kinase activity (GO:0004672). Of these, 623 were detected at the transcriptional level, with *MAPK17* showing the highest expression (SA-TPM = 537, File S1). Only 187 (~30%) had corresponding proteins identified in the proteomes of etiolated seedlings (Table 1), a transcript-to-protein ratio comparable to that observed for the entire transcriptome (~34.6%). Among the PK genes without detectable protein products, 14 showed high transcript abundance (SA-TPM > 50, Table 1), including six genes encoding calcineurin B-like (CBL)-interacting PKs: *CIPK1/7/12/15*, and SALT OVERLY SENSITIVE (SOS)3 interacting proteins *SIP3/4*, and a CPK-related protein (*CRK2*). Conversely, one PK gene (AT5G59680) encoding a Leucine-Rich Repeat Protein Kinase Family Protein (LRR-PK) was detected at the protein level despite having undetectable transcripts consistent with protein stability or synthesis at earlier developmental stages.

**Figure 4:**
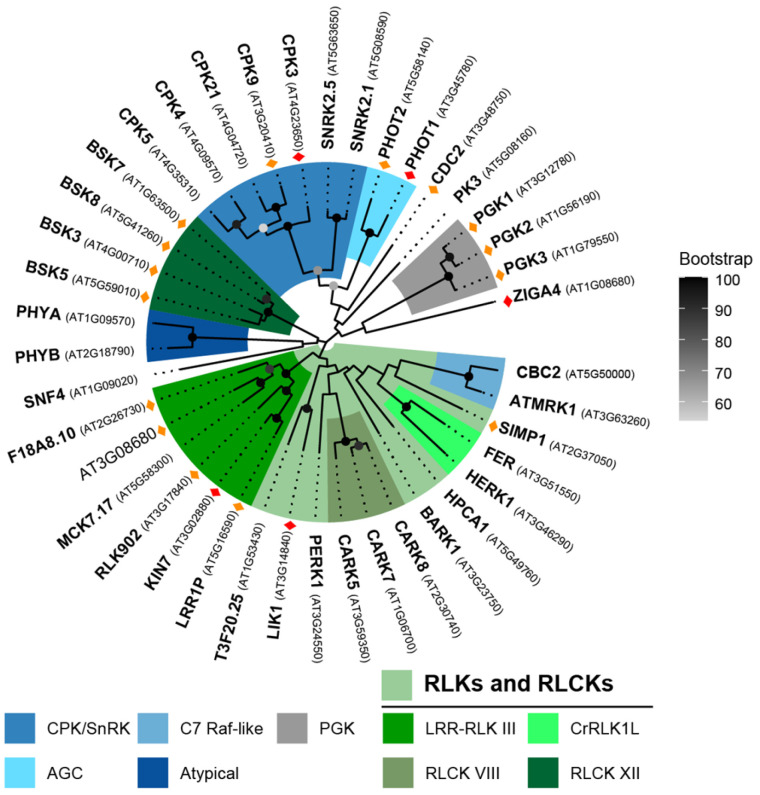
Phylogenetic analysis of 41 most abundant PKs (SA-prot > 50) detected in etiolated seedlings. The inferred phylogeny was based on full-length protein alignments. Prominent PK families are color-coded. Bootstrap support values > 50 (from 100 replicates) are shown at the nodes. Phosphorylated PKs are marked with (♦) or (♦) if detected in both or in one phosphoproteome, respectively. SNF4 (a regulatory subunit of SnRK1), the zinc finger ARF GAP-like protein ZIGA4, and both phytochromes are not canonical kinases but were included due to their annotation with kinase-related GO terms.

Of the 194 PKs identified in the proteome, 41 exhibited high protein abundance (Table 1), providing a snapshot of the PKs potentially active during skotomorphogenesis. Phylogenetic analysis (Figure 4) revealed clustering into three major branches. One monophyletic group comprises 14 receptor-like kinases (RLKs) and three receptor-like cytoplasmic kinases (RLCKs). Among the RLKs, six members belong to the LRR-III subfamily, while FERONIA (FER) and HERCULES RECEPTOR KINASE 1 (HERK1) are classified within the CrRLK1L subfamily. Two Raf-like kinases of the C7 subfamily cluster within the same branch. Another branch includes four brassinosteroid signaling kinases (BSKs; RLCK XII) and two phytochromes (PHYA and PHYB), in separate subgroups. The third branch contains various soluble kinases: seven members of the SnRK/CPK superfamily (CPK3/4/5/9/21 and SnRK2.1/2.5), two of the AGC family (PHOT1 and PHOT2), CDC2 from the CMGC family, and PK3, which is grouped distantly from three glycolysis-related phosphoglycerate kinases (PGKs).

Tissue expression analysis of the most abundant 41 PKs shows that they are ubiquitously expressed except for mature pollen. Transcriptomic analysis of seedlings exposed to different light treatments shows that most PKs do not undergo significant transcriptional changes in response to light (Figure S4).

In conclusion, etiolated seedlings exhibit selective expression of PKs, and those involved in calcium signaling emerge among the most abundant soluble kinases.

### 2.4. Phosphorylation Patterns of Protein Kinases in Etiolated Seedlings

**Figure 5:**
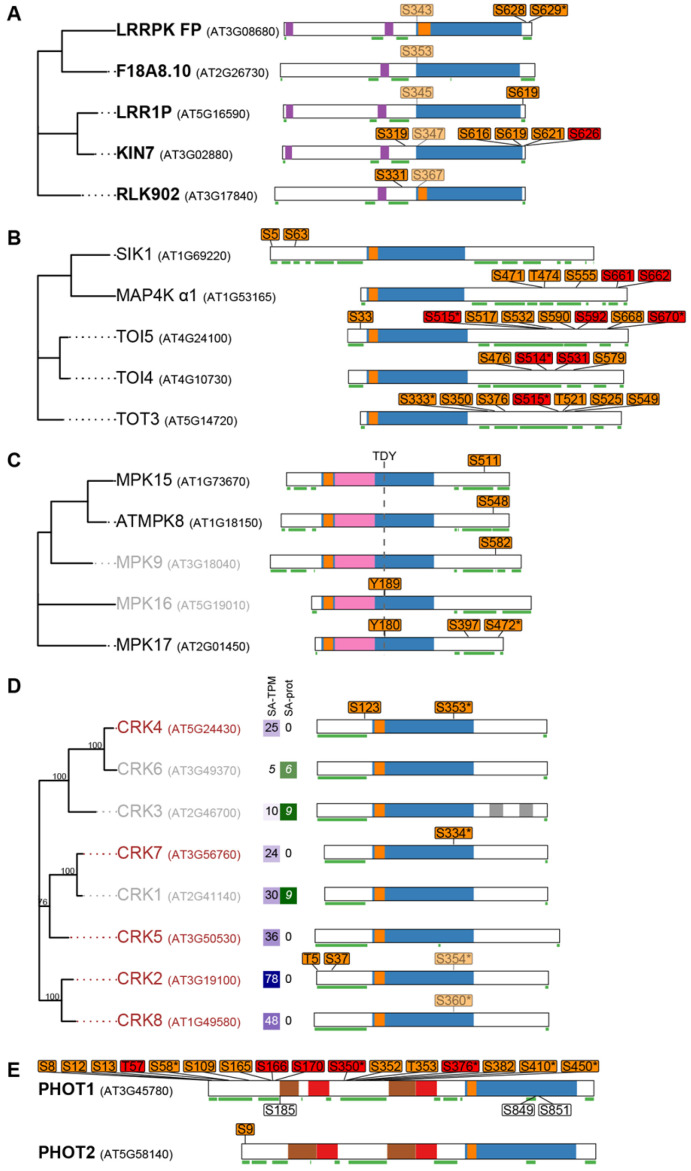
Phosphorylation patterns of LRR-III kinases (**A**), MAP4Ks (**B**), MAPKs (**C**), CRKs (**D**) and Phototropins (**E**). PK names in **bold**: high protein abundance; gray: uncertain presence; brown: not detected in any proteome. Domain architecture is based on ScanProSite and TMHMM predictions: ⏹ KD; ⏹ ATP binding site; ⏹ transmembrane helix; ⏹ MAPK domain; ⏹ EF hand; ⏹ PAS domain (Per-ARNT-Sim); ⏹ PAC (PAS-associated C-terminal) motif. Disordered regions, based on PrDOS predictions, are underlined in green. pS/T: phosphosites detected in both phosphoproteomes, pS/T/Y: in only one dataset. Non-exclusive phosphopeptides are displayed with transparency. (*) Phosphorylations in an RxxS context. The ATP-binding site was absent in F18A8.10, LRR1, and KIN7. LRR1 and KIN7 belong to the atypical non-RD kinase subgroup and lack in vitro kinase activity [43]. (**D**) SA-TPM and SA-prot values are indicated for CRKs. (**E**) In PHOT1, no phosphorylated residues of S185, S849 and S851 are indicated.

Out of the 41 abundant kinases, 20 (48.8%) were phosphorylated (Figure 4). Phosphorylated PKs were significantly overrepresented in etiolated seedlings (χ^2^ = 36.4, *p* < 0.00001; Table 1), and the proportion increased further among highly abundant PKs, with 85.7% of those with SA-prot > 80 being phosphorylated; far above the average phosphorylation rate for proteins of similar abundance (χ^2^ = 7.38, *p* = 0.0066). In addition to these 20 phosphorylated PKs, 115 other PKs were identified in the phosphoproteome, including those with detectable and undetectable protein levels (Figure S5). Phosphorylation events were particularly enriched in specific subfamilies, enabling the formulation of multiple hypotheses that must be tested in future experimental studies. LRR-III kinases shared a conserved phosphosite in subdomain I, and three members were also phosphorylated in the CT (Figure 5A). CT phosphorylation can create docking sites for downstream substrates in RLK signaling pathways [38] and modulate substrate phosphorylation, as shown for BRI1 [39]. In line with Mitra et al. (2015) [40], CT phosphosites in these RLKs may facilitate substrate recognition. The seven BSKs identified displayed conserved phospho-serine residues (pS, Figure S6A) that are essential for activation [41]. Furthermore, phosphorylated versions of their proposed targets, BSL1 and BSL2 [42], were also detected (Figure S6A). The five MAP4Ks showed multiple NT or CT phosphorylation sites; in particular, TOT3, TOI4, and TOI5 contained several RxxS phosphoserines detected in both phosphoproteomes (Figure 5B).

The phosphokinome uncovered additional features. All identified MAPKs (8, 9, 15, 16, and 17) belong to group D1, characterized by a TDY activation motif, absence of a CD domain, and an extended CT region. CD domains contain acidic residues essential for interaction with a basic amino acid cluster in MAPKKs [44]. The pSs in the CT of MAPK8/9/15 and 17 (Figure 5C) may provide an alternative MAPKKs docking mechanism. Among MAPKKs, only MKK2 (At4G29810) was confidently detected in the phosphokinome, while four others were present in the proteome at low abundance. Five casein kinase 1-like (CKL) kinases shared conserved phosphosites in their CTs, a region responsible for determining substrate specificity (Figure S6B).

Four CPK-Related Kinases (CRK2/4/7/8) were detected in the transcriptome and phosphokinome, but not in the proteome (SA-prot = 0), suggesting that phosphorylation may negatively regulate protein stability during skotomorphogenesis (Figure 5D). These CRKs shared phosphosites in a similar RxxS context ([A/G]R[T/S]EpS[A/G]IFR) within the catalytic domain. By contrast, CRK1/3/6, with SA-prot values between 6.2 and 9, were not phosphorylated.

Plasma membrane-associated PHOTs are blue light receptors that mediate phototropism [45] and calcium signaling [46]. PHOTs contain two NT LOV domains (PAC-PAS in Figure 5E) separated by a hinge region and a C-terminal KD. Multiple phosphorylation sites have been reported, but only phosphosites in the activation loop are essential for PHOT1 function [47,48,49]. As observed in Figure 5E, PHOT1 showed extensive phosphorylation across its N-terminal and hinge regions, whereas PHOT2 displayed a single phosphosite in its N-terminal domain. Several phosphosites previously described as blue light–dependent [47,50,51,52] were detected in darkness and in other developmental contexts, suggesting broader regulatory roles. Many of these sites occur in an RxxS motif and are located within the hinge region, where phosphorylation has been associated with 14-3-3 protein binding [47,50,53]. Consistently, multiple 14-3-3 proteins were detected at high abundance in etiolated seedlings (Table S4).

In summary, we observed distinct phosphorylation patterns across PK families, including sites potentially linked to kinase activation (BSKs), substrate recognition (LRR-III RLKs and CKLs), protein–protein interactions (PHOT1–14-3-3, D1 MAPKs-MAPKKs), or protein degradation (CRKs). Several phosphosites occurred in the RxxS motif, a known target of CPKs. Given their abundance at this developmental stage, we examined the CPK family in detail.

### 2.5. CPK3 and CPK9 Are Prominent CPKs in Dark-Grown Seedlings

**Figure 6:**
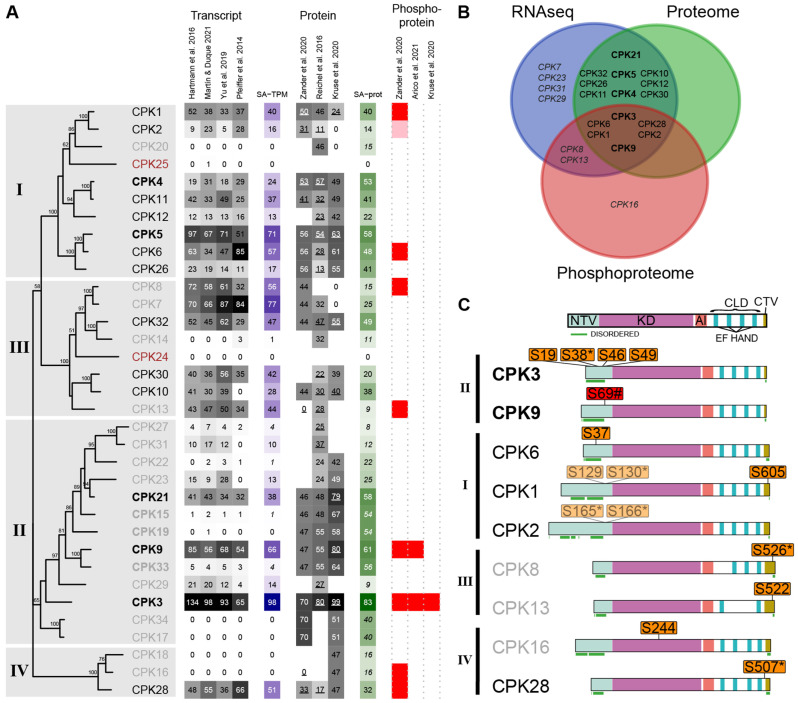
Meta-analysis of AtCPK transcripts, proteins, and phosphoproteins in dark-grown seedlings. (**A**) Phylogenetic tree of AtCPKs with four subgroups indicated. CPKs in **bold**: highly abundant; gray: uncertain detection; brown: not detected. Transcript and protein abundances are shown as heat maps of normalized S-TPM and S-prot values (gray scale, relative to the highest value), with SA-TPM or SA-Prot values on the right (blue or green scales, respectively). Numbers in *italics* in SA-TPM/SA-prot: uncertain or ambiguous detection; underlined in SA-prot: exclusive peptides. Presence in phosphoproteomes is indicated by red (exclusive phosphopeptides) or pink boxes (ambiguous phosphopeptides). Dataset authors are indicated in column headers [29,30,31,32,33,34,35,36]. (**B**) Venn diagram of AtCPKs detected in transcriptome, proteome, and phosphoproteome datasets. (**C**) Domain structure of AtCPKs: NTV (N-terminal variable), KD (kinase domain), AI (autoinhibitory), CLD (calmodulin-like), CTV (C-terminal variable). Disordered regions are underlined in green. pS: detected in both phosphoproteomes, pS: in only one dataset. Non-exclusive phosphopeptides are displayed with transparency. (*) indicates phosphorylations in an RxxS context. (#) S69 was detected by Zander et al. (2020, both replicates) [34] and by Arico et al. (2021, one replicate) [36].

Meta-analysis of the CPK family showed that 21 of the 34 *AtCPK* members were expressed above the transcript threshold, while only 15 were detected at the protein level in etiolated seedlings (Figure 6A,B). The most abundant proteins included AtCPK3, AtCPK9, and AtCPK21 (subgroup II), AtCPK4, AtCPK5, and AtCPK6 (subgroup I), and AtCPK32 (subgroup III). Although *AtCPK7* and *AtCPK8* transcripts were highly abundant, their proteins could not be reliably detected (Figure 6A). Most CPKs were ubiquitously expressed across tissues, excluding mature pollen, and were also detected in imbibed seeds, indicating their presence in the nascent seedling. *AtCPK3*, *AtCPK5*, and *AtCPK32* maintained consistently high expression across tissues (Figure S7). Transcriptomic datasets from dark-grown seedlings exposed to white, blue and red light pulses (1 to 6 h) showed no major changes in CPK expression during dark-to-light transitions, suggesting that the same CPK set mediates calcium sensing during early development (Figure S7).

CPKs are known to undergo autophosphorylation, which modulates their activity [54,55,56,57,58,59,60,61]. We identified phosphorylated forms of AtCPK1, AtCPK2, AtCPK3, AtCPK6, AtCPK8, AtCPK9, AtCPK13, AtCPK16, and AtCPK28 (Figure 6B,C). However, AtCPK8, 13 and 16 did not meet the criteria for bona fide phosphoproteins, and *CPK16* transcripts were not detected. Phosphorylated forms of AtCPK3 and AtCPK9 were detected in both phosphoproteomes and Kruse et al. (2020) [35] also identified AtCPK3 as a phosphoprotein.

Most phosphosites were located within the NT domains of AtCPK1, AtCPK2, AtCPK3, AtCPK6, and AtCPK9, while additional phosphosites were identified in the CT domains of AtCPK1, AtCPK8, AtCPK13, and AtCPK28. NORSp analysis indicated that these NT and CT regions contain long stretches of disordered structure (NORS region, Figure 6C). Several CPKs (AtCPK1/2/3/8/28) harbored pSs in the RxxS context (Figure 6C). A χ^2^ test comparing the frequency of phosphorylated RxxS motifs in the overall phosphoproteome (13.5%, 381 out of 2827) with that in AtCPKs (45.5%, 5 out of 11) revealed a significant enrichment among AtCPKs (χ^2^ = 8.7, *p* < 0.005), supporting the occurrence of intra- or intermolecular autophosphorylation events within this family. Because RxxS motifs can be recognized by multiple Ca^2+^-dependent kinases, this enrichment could be interpreted as indicative of family-level regulatory potential.

Several proteins annotated in the PhosPhAt 4.0 database (University of Tübingen, Tübingen, Germany; https://phosphat.uni-hohenheim.de/) as putative CPK targets were detected in the phosphoproteomic datasets of etiolated seedlings. This information is presented as a reference resource to facilitate future targeted experimental analyses rather than as evidence of direct kinase–substrate relationships (Figure S8). RxxS motifs were also significantly enriched among phosphorylated microtubule-associated proteins (MAPs) (MAPs, χ^2^ = 5, *p* < 0.05; Table 1). MAPs are highly represented (χ^2^ = 97.6, *p* < 0.00001) and phosphorylated (χ^2^ = 97.4, *p* < 0.00001) at this developmental stage [62]. Together, these results highlight MAPs as a prominent group of phosphorylated proteins in etiolated seedlings and suggest that they represent a promising focus for future studies on calcium-dependent signaling.

Our analysis indicates that AtCPK3 and AtCPK9 were consistently detected at the transcript, protein, and phosphoprotein levels in etiolated seedlings, emerging as interesting components of calcium signaling cascades during this developmental stage.

### 2.6. Most PPs Are Expressed and Display Broad Functional Diversity in Dark-Grown Seedlings

Protein phosphatases balance kinase activity: of the 152 PPs present in the Arabidopsis genome, 124 were transcriptionally active and 45 were detected with high confidence in the proteome of etiolated seedlings (Table 1, Figure S9). Most of these PPs exhibited a constitutive expression pattern across all tissues, including mature pollen (Figure S9). Interestingly, while the frequency of PKs (18.4%) closely matched the genome-wide protein detection rate (18.3%), PPs (29.6%) were significantly overrepresented (*p* = 0.00034; χ^2^ = 12.8) (Table 1). This enrichment was primarily attributable to a significantly higher transcription rate (81.6%) compared to the genome-wide average (52.9% χ^2^ = 49.93, *p* < 0.00001), whereas their translation efficiency (36.3%) did not significantly differ from that of the full transcriptome (34.6%).

Only one PP, Release of DOrmancy 5/Delay Of Germination 18 (RDO5/DOG18) was present in the proteome despite having undetectable transcripts, while three had high transcript levels (SA-TPM > 50) but no detected protein: *AtPGPP1* involved in phosphatidylglycerol biosynthesis and photosynthetic function [63], a thylakoid associated phosphatase, and *PP2C.D6* that inhibits the Na^+^/H^+^ antiporter activity of SALT OVERLY SENSITIVE (SOS1) under non-salt-stress conditions (Table 1, File S1). Of the 21 phosphosites identified across these nine PPs (Figure S9), most mapped to intrinsically disordered regions and six matched the RxxS consensus motif (Figures S6A and S10). Phosphorylation at this motif was significantly overrepresented (*p* < 0.01, χ^2^ = 7) compared with the global phosphoproteome of etiolated seedlings (Table 1).

In summary, PPs show higher transcript and protein expression than the genome-wide average, with members of multiple families highlighting their broad regulatory potential in etiolated seedlings. Phosphorylation of PPs suggests they are themselves regulated post-translationally.

## 3. Discussion

In this study, we developed OmicIntegrator, which enables direct cross-layer comparisons and facilitates the systematic identification of concordant and discordant regulatory patterns by harmonizing data scales and applying consistent operational criteria. Rather than providing an exhaustive or temporally resolved molecular inventory, OmicIntegrator was designed to contextualize multi-omic signals within a defined developmental stage. Overall, our analyses do not establish causal regulatory relationships but instead provide a systems-level resource that integrates signaling components across multiple omic layers. By contextualizing phosphorylation events, protein abundance, and transcriptional activity within a single framework, OmicIntegrator enables the prioritization of candidate regulators and pathways for targeted experimental validation. This work lays a foundation for future studies aimed at dissecting the molecular mechanisms governing skotomorphogenesis.

As a case study, we applied this framework to omic datasets from etiolated Arabidopsis seedlings. This analysis revealed widespread divergence between transcript abundance, protein accumulation, and phosphorylation status, underscoring the value of multi-omic integration for interpreting regulatory complexity during early development. For instance, protein detection in the absence of corresponding transcripts is consistent with protein persistence from earlier developmental stages and reflects the reliance of etiolated seedlings on pre-existing reserves (Figure S3). Conversely, several photosynthesis-related genes exhibited high transcript levels but lacked detectable proteins, a pattern consistent with post-transcriptional and/or post-translational regulation that prevents premature accumulation of photosynthetic machinery in darkness. Importantly, these patterns were observed despite differences in experimental parameters among datasets (Table S5), since darkness is the dominant factor shaping the biological response. Finally, we should consider that the absence of a detectable protein signal does not necessarily imply a lack of translation or biological relevance but rather indicates that protein abundance falls below current detection thresholds at this developmental stage.

The broad representation of PKs and PPs in etiolated seedlings provides a global view of the signaling components available during skotomorphogenesis. Notably, FER and HERK1 emerged as abundant RLKs consistent with their reported roles in cell wall integrity sensing and growth regulation [64]. The presence of photoreceptors such as PHYA, PHYB, and phototropins further indicates that seedlings are pre-equipped to perceive light cues prior to emergence. Importantly, most PKs and PPs showed limited transcriptional changes upon light exposure (Figures S4 and S9), supporting a model in which signaling transitions from skotomorphogenesis to photomorphogenesis are largely mediated through post-translational mechanisms rather than transcriptional reprogramming.

Placing individual signaling pathways within this multi-omic framework provides a physiological context rather than functional validation and enables the formulation of multiple hypotheses. Members of the LRR-III subfamily of RLKs, as well as BSKs and CPKs, were among the most abundant and frequently phosphorylated PKs, highlighting them as prominent components of the signaling landscape in etiolated seedlings (Figure 4). In particular, phosphorylation of BSKs at known activation-associated sites, together with phosphorylation of BSL1 and BSL2 (Figure S6A), is consistent with active brassinosteroid signaling during hypocotyl elongation. While BSKs are likely contributors to BSL phosphorylation, alternative upstream kinases cannot be excluded [65], emphasizing the integrative rather than mechanistic nature of these observations. CPK3 and CPK9 repeatedly emerged across transcriptomic, proteomic, and phosphoproteomic layers (Figure 6, Figure S7), identifying them as recurrent candidates within calcium-dependent signaling networks in darkness. According to PhosPhAt 4.0, CPK3 and CPK9 contain 14 reported autophosphorylation sites, most within their NT domains. However, only a subset was phosphorylated in dark-grown seedlings (Figure 6C), suggesting that the phosphorylation status of CPKs may vary depending on the plant’s developmental stage and/or environmental cues.

Phosphorylation of PHOT1 at RxxS motifs located in the hinge region, previously associated with 14-3-3 (GRF) binding [47,50,53], was detected under dark conditions. GRFs, which recognize RxxS phosphosites and modulate protein stability, localization, and complex assembly [66], were abundant and partially phosphorylated in etiolated seedlings. The co-occurrence of phosphorylated PHOT1, GRFs, and CPK-associated motifs suggests potential regulatory interactions operating prior to light exposure. However, these associations should be interpreted as contextual correlations within the phosphoproteome rather than evidence of direct regulation.

Protein phosphatases were also broadly represented and, in some cases, phosphorylated at RxxS motifs, particularly within regulatory subunits of PP2A-like complexes (Figure S10). Since phosphatase specificity is largely determined by regulatory subunits, such modifications may influence complex composition or activity. Similar regulatory principles have been described in other plant systems, supporting the idea that phosphatases themselves participate dynamically in phosphorylation-dependent signaling networks. In addition, the overrepresentation of phosphorylated MAPs (Arico et al., 2024 [62], Table 1) highlights the dynamic regulation of the cytoskeleton during early seedling development, with RxxS motif enrichment (Table 1) consistent with calcium-dependent signaling as a potential contributing layer. Among the kinase consensus motifs analyzed, only RxxS sites showed significant enrichment in the categories analyzed in the phosphoproteome (PKs, PPs, MAPs), whereas proline-directed and acidic CK2 motifs were not overrepresented (Table 1), indicating motif-specific rather than global phosphorylation bias.

Given its agnostic design, this framework can be applied to test hypotheses in different organisms, depending on the datasets provided. Importantly, OmicIntegrator uses adjustable thresholds rather than fixed constraints, allowing the workflow to be adapted to different biological questions, data qualities, and experimental designs.

## 4. Materials and Methods

### 4.1. Computational Pipeline Developed

A program implemented in C# (.NET 9; Microsoft, Redmond, WA, USA, open source) handles data ingestion into a relational SQLite (SQLite Consortium, Hipp, Wyrick & Company, Charlotte, NC, USA) database and is operated via a console interface. Data from TAIR (GFF files, chromosomal FASTA sequences, and GO annotations, https://www.arabidopsis.org/), as well as from ScanProsite (Swiss Institute of Bioinformatics, Lausanne, Switzerland, https://prosite.expasy.org/scanprosite/) and TMHMM-2.0 [67] (Technical University of Denmark, Lyngby, Denmark, https://services.healthtech.dtu.dk/services/TMHMM-2.0/, accessed on 20 October 2025), and NOn-regular secondary structure (NORSp; PrDOS, European Molecular Biology Laboratory, Heidelberg, Germany, https://prdos.hgc.jp/cgi-bin/top.cgi) were loaded into the database. For transcriptomic input, the pipeline converts raw RNAseq counts and RPKM values into TPM. Proteomic and phosphoproteomic matrices were mapped to their corresponding genes based on peptide sequences when available.

Quantitative normalization was performed in R using linear regression models, with the results written back into the SQLite database. Throughout the workflow, conservative and consistent operational criteria were applied to enable robust comparisons across heterogeneous datasets. These criteria are intended to reflect confidence in detection rather than absolute biological presence or absence. Although applied here to etiolated *Arabidopsis thaliana* seedlings as a case study, the OmicIntegrator framework is organism and condition-agnostic and can be adapted to other biological systems, developmental stages, or environmental conditions.

Data visualization was also managed in R (version 4.5.1; R Foundation for Statistical Computing, Vienna, Austria), including heatmaps, phosphorylation diagrams, and phylogenetic trees (from NWK files), as well as all figures included in this work. Extracting and plotting any subset of genes required only minor R handling. To ensure reproducibility and facilitate reuse by the community, all source code, scripts, and datasets used in this study are available on GitHub (https://github.com/ifgitman/OmicIntegrator; accessed on 20 October 2025).

### 4.2. Standardization of Datasets Used in This Study

We analyzed public RNA-seq [29,30,31,32] and proteomic [33,34,35] data performed with Arabidopsis Col-0 or Ler seedlings grown under dark conditions for 2 to 6 days in half-strength MS at 22 °C (Table S5). To normalize RNAseq data across the four experiments, TPM values for each of the 27,533 genes were first averaged. Then, a linear regression on a logarithmic scale was performed for each experiment (ln y) using the previously calculated average (ln x) as the reference (Figure S1). This regression-based transformation adjusted the original data, allowing for the computation of a Standardized TPM (S-TPM) for each gene in each experiment as well as a Standardized Average TPM (SA-TPM).

To enable robust cross-study comparisons and minimize technical noise inherent to large-scale meta-analyses, we applied conservative operational thresholds. Genes were considered transcribed when S-TPM values were >5 in at least three experiments and considered below the detection thresholds when S-TPM values were <1 in at least three experiments. Genes with intermediate values were classified as ambiguous. Highly abundant transcripts were defined as those with SA-TPM > 50. These thresholds were intentionally chosen to prioritize reproducibility and cross-dataset consistency and therefore delineate a high-confidence core of transcriptional activity, rather than a complete regulatory landscape.

Protein abundance was estimated by summing the abundance of all peptides corresponding to each protein. To standardize the proteomic data from the three datasets, a linear regression on a logarithmic scale was performed between the protein abundance values in each experiment (ln y) and the corresponding SA-TPM values (ln x) for genes with detectable transcript levels (Figure S2). Standardized protein abundance (S-prot) equal to 0 was assigned to proteins that were not detected. The fitted values were then averaged to compute a Standardized Average protein abundance (SA-prot) for each gene. Proteins were considered present when detected in at least two independent proteomes, while SA-prot > 50 was used to classify highly abundant proteins. As for transcriptomic data, these criteria were selected to emphasize robust, reproducible signals across heterogeneous datasets, acknowledging that low-abundance or condition-specific proteins with biological relevance may be underrepresented.

We also analyzed phosphoproteomic datasets from Arabidopsis seedlings grown in darkness [34,36]. Phosphopeptides whose abundance exceeded the threshold in at least two replicates within each dataset were mapped to their corresponding proteins. Additionally, we queried the dataset from Kruse et al. (2020) [35], which reports post-translational protein modifications during this developmental stage.

AtPKs were filtered using the Gene Ontology (GO) ‘protein kinase activity’ (GO:0004672) term, AtPPs were filtered using terms ‘protein phosphatase’, or GO:0004721 ‘phosphoprotein phosphatase activity’ and GO:1903293 ‘phosphatase complex’ and manually curated, and microtubule-associated proteins (AtMAPs) were identified as described in Arico et al. (2024) [62]. CPK targets reported by PhosPhAt 4.0 experimental data were analyzed. Phylogenetic analyses were conducted using MEGA-X [68] (Molecular Evolutionary Genetics Analysis; Center for Evolutionary Medicine and Informatics, The Biodesign Institute, Arizona State University, Tempe, AZ, USA). To identify PK recognition motifs within the Arabidopsis phosphoproteomes of etiolated seedlings, we searched for known phosphorylation consensus sequences reported by Wang et al. (2013) [37], focusing on LxRxxS and RxxS for CPKs and Ca^2+^/CaMPKs; [S/T]P and Px[S/T]P for GSK-3, CDKs, and MAPKs (proline-directed motifs); and Sx[D/E], SDx[D/E], SDxED, and SExE for CK2 activity (acidic motifs). We analyzed their distribution among phosphopeptides to infer kinase activities.

Expression data for selected AtPKs, AtPPs, and members of the AtCPK family across different tissues were obtained from the Arabidopsis eFP Browser (Developmental Map dataset). In addition, their expression in Arabidopsis seedlings exposed to white, blue, and red light was evaluated using RNA-seq datasets from Yu et al. (2019) [32] and Hartmann et al. (2016) [30].

## 5. Conclusions

We developed OmicIntegrator to standardize and integrate transcriptomic, proteomic, and phosphoproteomic datasets and used *A. thaliana* etiolated seedlings as a case study focusing on reversible phosphorylation. Arabidopsis genome annotations were preloaded for this study, so this pipeline can be used directly by anyone working with this model plant. This integrative framework allowed us to gain deeper insight into the gene expression, protein abundance, and phosphorylation patterns that underlie the etiolated growth program and to formulate hypotheses to be tested in future studies. Moreover, this family-wide analysis revealed relationships and regulatory trends that remain obscured when only a single gene or the entire genome is examined. The results include findings that validate previous reports as well as novel or divergent observations. This meta-analytic, multi-omics approach provides a means to contextualize genes of interest in a chosen developmental and/or physiological context.

## Figures and Tables

**Table 1:** Summary of annotated genes, transcripts, and proteins in dark-grown *Arabidopsis thaliana* seedlings.

		All Genes			Kinases			Phosphatases			CPKs			MAPs		
			%			%			%			%			%	
		No.	Genes	Prot.	No.	Genes	Prot.	No.	Genes	Prot.	No.	Genes	Prot.	No.	Genes	Prot.
Genes	annotated genes	27,533	100.0		1055	100.0		152	100.0		34	100.0		1494	100.0	
	transcripts	14,564	52.9		623	59.1		**124**	**81.6**		21	61.8		**952**	**63.7**	
	proteins	5043	18.3	100.0	194	18.4	100.0	**45**	**29.6**	100.0	15	44.1	100.0	**427**	**28.6**	100.0
	proteins w/ transcripts	4777	17.4	94.7	187	17.7	96.4	43	28.3	95.6	15	44.1	100.0	409	27.4	95.8
	proteins w/o transcripts *	46	0.2	0.9	1	0.1	0.5	1	0.7	2.2	0	0.0	0.0	2	0.1	0.5
	SA-TPM ≥ 50	3692	13.4	73.2	113	10.7	58.2	28	18.4	62.2	7	20.6	46.7	205	13.7	48.0
	high transcript, w/o protein	777	2.8	15.4	14	1.3	7.2	3	2.0	6.7	0	0.0	0.0	14	0.9	3.3
	SA-prot ≥ 50	1596	5.8	31.6	41	3.9	21.1	13	8.6	28.9	5	14.7	33.3	152	10.2	35.6
	phosphoproteins	1019	3.7	20.2	**74**	7.0	**38.1**	9	5.9	20.0	6	17.6	40.0	**174**	11.6	**40.7**
	multiphosphorylated	556	2.0	11.0	39	3.7	20.1	7	4.6	15.6	3	8.8	20.0	111	7.4	26.0
Phospho sites	total	2827	100.0		183	100.0		21	100.0		11	100.0		595	100.0	
	RxxS	381	13.5		33	18.0		**7**	**33.3**		**5**	**45.5**		**101**	**17.0**	
	Acidic	554	19.6		33	18.0		9	42.9		0	0.0		131	22.0	
	Pro-d	653	23.1		41	22.4		4	19.0		2	18.2		132	22.2	

The table presents the total number of annotated genes and their representation at the transcript and protein levels. Counts for PKs, PPs, CPKs, and MAPs are listed. Percentages (%) relative to the annotated genes or detected proteins in each gene set are shown. Phosphosites detected in the different protein sets were classified as RxxS (Ca^2+^/CaMPKs and CPKs), acidic (CK2) and Pro-d (GSK-3, CDKs and MAPKs). Categories that are significantly overrepresented (χ^2^ test; *p* < 0.05) compared to the whole genome, proteome, or phosphoproteome, are highlighted in bold. * proteins with uncertain transcript presence are not considered.

## Data Availability

Data is available at GitHub (https://github.com/ifgitman/OmicIntegrator; accessed on 20 October 2025).

## Supplementary Materials

The following supporting information can be downloaded at https://www.mdpi.com/article/10.3390/plants15020334/s1. File S1: The genome-wide integration output for 27,533 *Arabidopsis thaliana* genes. Table S1. GO term analysis of 288 genes with very high transcript levels (SA-TPM > 100), but no detectable protein (SA-prot = 0), revealed a significant enrichment in photosynthesis-related genes. Table S2. GO term analysis of 46 proteins with undetectable transcript levels revealed a significant enrichment in post-embryonic development, carbohydrate catabolism, and nutrient reservoir activity. Table S3. List of 37 phosphoproteins with high transcript abundance but no detectable protein levels in any of the proteomic datasets. Table S4. List of the 13 genes encoding 14-3-3 proteins (GRFs). Table S5. Datasets analyzed in this work. Figure S1. Normalization of RNA-seq data from four *Arabidopsis thaliana* dark-grown seedlings datasets. Figure S2. Normalization of proteomic data from three *Arabidopsis thaliana* dark-grown seedlings datasets. Figure S3. Tissue expression pattern of the 46 proteins with undetectable transcript levels. Figure S4. Tissue-specific expression and light-mediated expression fold of the 41 PKs are abundantly expressed in etiolated seedlings. Figure S5. Phylogenetic analysis of 135 phosphorylated PKs detected in etiolated seedlings. Figure S6. Phosphorylation patterns in BSKs, BSLs, and CKLs. Figure S7. Tissue-specific expression and light-mediated expression fold of the different members of the AtCPK family. Figure S8. CPK targets reported in the PhosPhAt 4.0 database and detected in the phosphoproteome of etiolated seedlings. Figure S9. Tissue-specific expression and light-mediated expression fold of the 45 PPs expressed in etiolated seedlings. Figure S10. Phosphorylation patterns in PPs.

## Abbreviations

The following abbreviations are used in this manuscript:

CPK	calcium-dependent protein kinase
CT	C-terminal domain
GO	gene ontology
MAPS	microtubule-associated proteins
NT	N-terminal domain
PK	protein kinase
PP	protein phosphatase
pS	phosphoSerine
SA-prot	standardized average protein abundance
SA-TPM	standardized average TPM
TPM	transcripts per million
