# Supplementary material for: OmicIntegrator: A Simple and Versatile Tool for Meta-Analysis
Source: Plants (Basel). 2026 Jan 22;15(2):334. doi: 10.3390/plants15020334 (PMC12845079; doi:10.3390/plants15020334)
Supplement: Supplementary file 1 [file plants-15-00334-s001.zip › Figure S1.pdf]

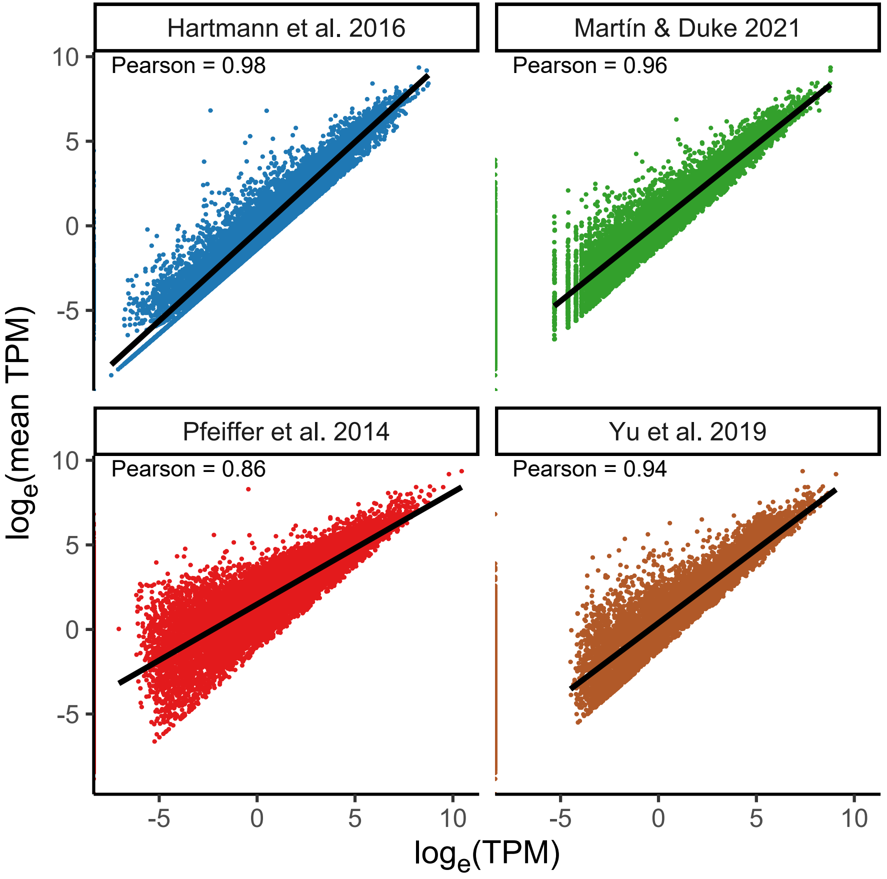

Figure S1. Normalization of RNA-seq data from four Arabidopsis dark-grown seedlings datasets. Logarithmic linear regressions for each dataset, plotting the natural logarithm of the mean TPM value per gene across datasets (y-axis) against the natural logarithm of the TPM value for the same gene in the corresponding dataset (x-axis). Pearson correlation indexes are indicated.
