# Supplementary material for: OmicIntegrator: A Simple and Versatile Tool for Meta-Analysis
Source: Plants (Basel). 2026 Jan 22;15(2):334. doi: 10.3390/plants15020334 (PMC12845079; doi:10.3390/plants15020334)
Supplement: Supplementary file 1 [file plants-15-00334-s001.zip › Figure S10.pdf]

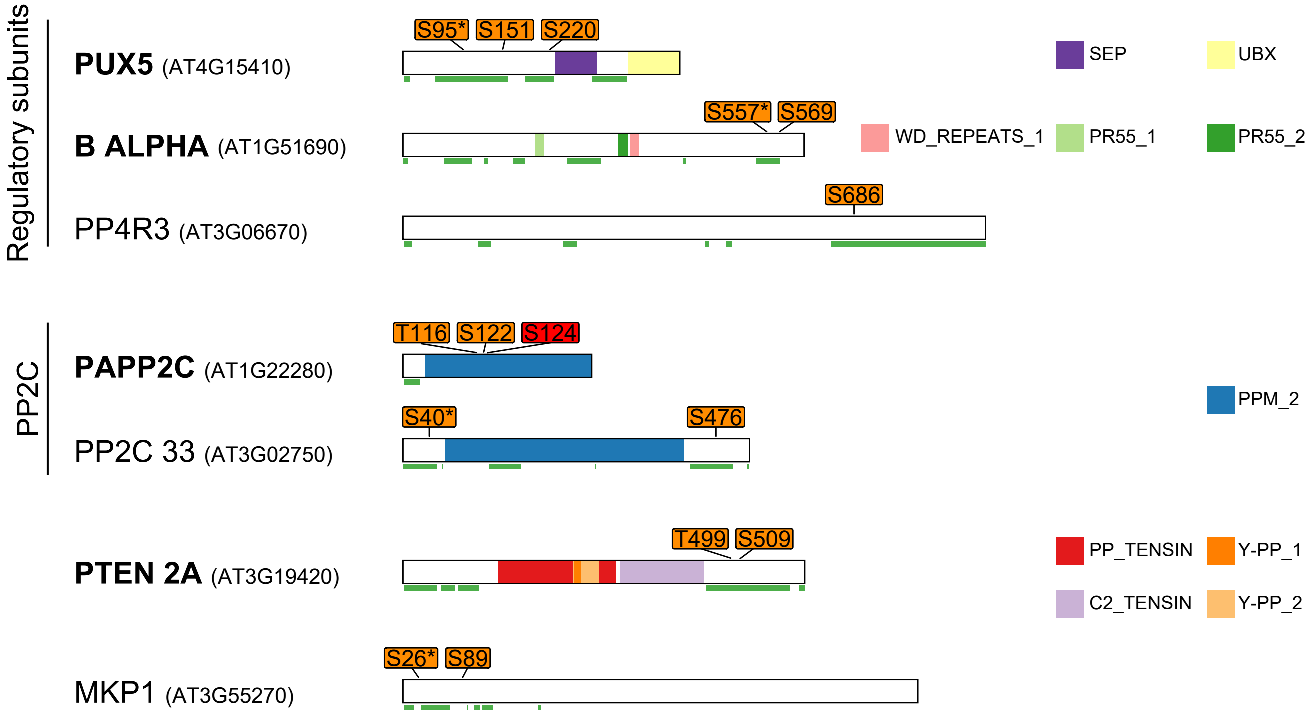

Figure S10. Phosphorylation patterns in PPs. PP names in **bold**: high protein abundance. Domain architecture is depicted based on ScanProSite and TMHMM predictions. Disordered regions, based on PrDOS predictions, are underlined in green. pS: detected in both phosphoproteomes, pS/pT: detected in only one dataset. (*) Phosphorylations in an RxxS context.
