# Supplementary material for: OmicIntegrator: A Simple and Versatile Tool for Meta-Analysis
Source: Plants (Basel). 2026 Jan 22;15(2):334. doi: 10.3390/plants15020334 (PMC12845079; doi:10.3390/plants15020334)
Supplement: Supplementary file 1 [file plants-15-00334-s001.zip › Figure S2.pdf]

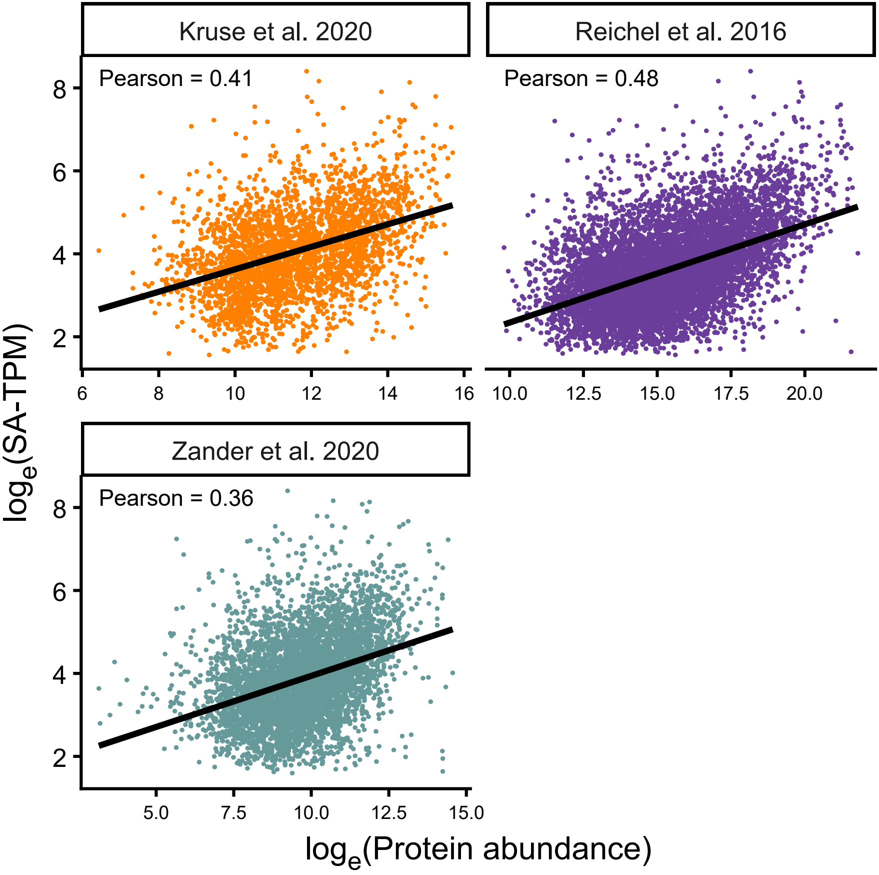

Figure S2. Normalization of proteomic data from three Arabidopsis dark-grown seedlings datasets. A linear fit on a logarithmic scale was performed between the protein abundance values in each experiment and their corresponding SA-TPM for genes with detectable transcript levels. Pearson correlation indexes are indicated.
