# Supplementary material for: OmicIntegrator: A Simple and Versatile Tool for Meta-Analysis
Source: Plants (Basel). 2026 Jan 22;15(2):334. doi: 10.3390/plants15020334 (PMC12845079; doi:10.3390/plants15020334)
Supplement: Supplementary file 1 [file plants-15-00334-s001.zip › Figure S3.pdf]

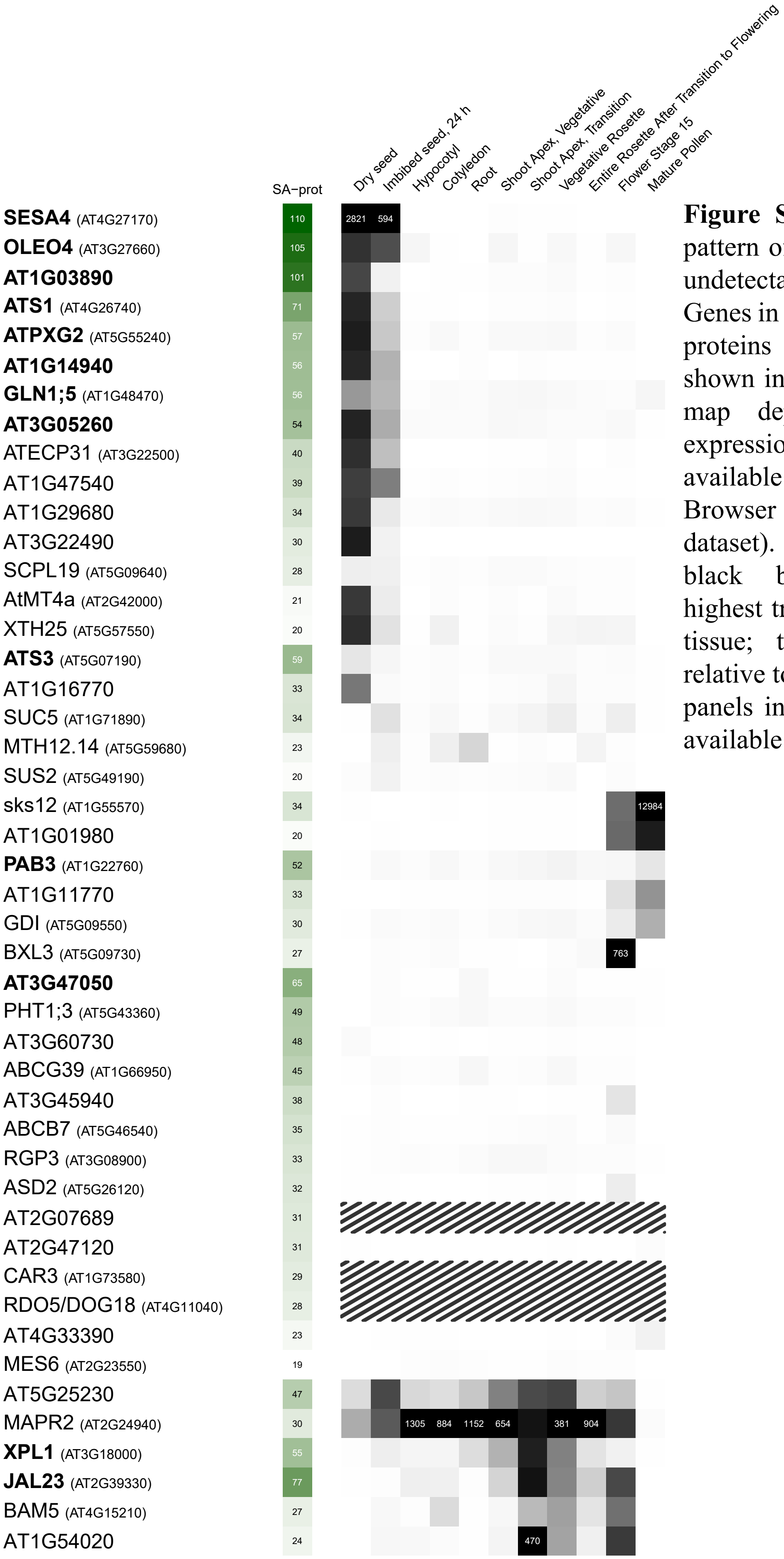

Figure S3. Tissue expression pattern of the 46 proteins with undetectable transcript levels. Genes in **bold**: highly abundant proteins (SA-prot values shown in a green scale). Heat-map depicting their tissue expression according to data available at Arabidopsis eFP Browser (Developmental Map dataset). Numbers inside the black boxes indicate the highest transcript level in each tissue; the gray scales are relative to these values. Striped panels indicate that no data is available.
