# Supplementary material for: OmicIntegrator: A Simple and Versatile Tool for Meta-Analysis
Source: Plants (Basel). 2026 Jan 22;15(2):334. doi: 10.3390/plants15020334 (PMC12845079; doi:10.3390/plants15020334)
Supplement: Supplementary file 1 [file plants-15-00334-s001.zip › Figure S6.pdf]

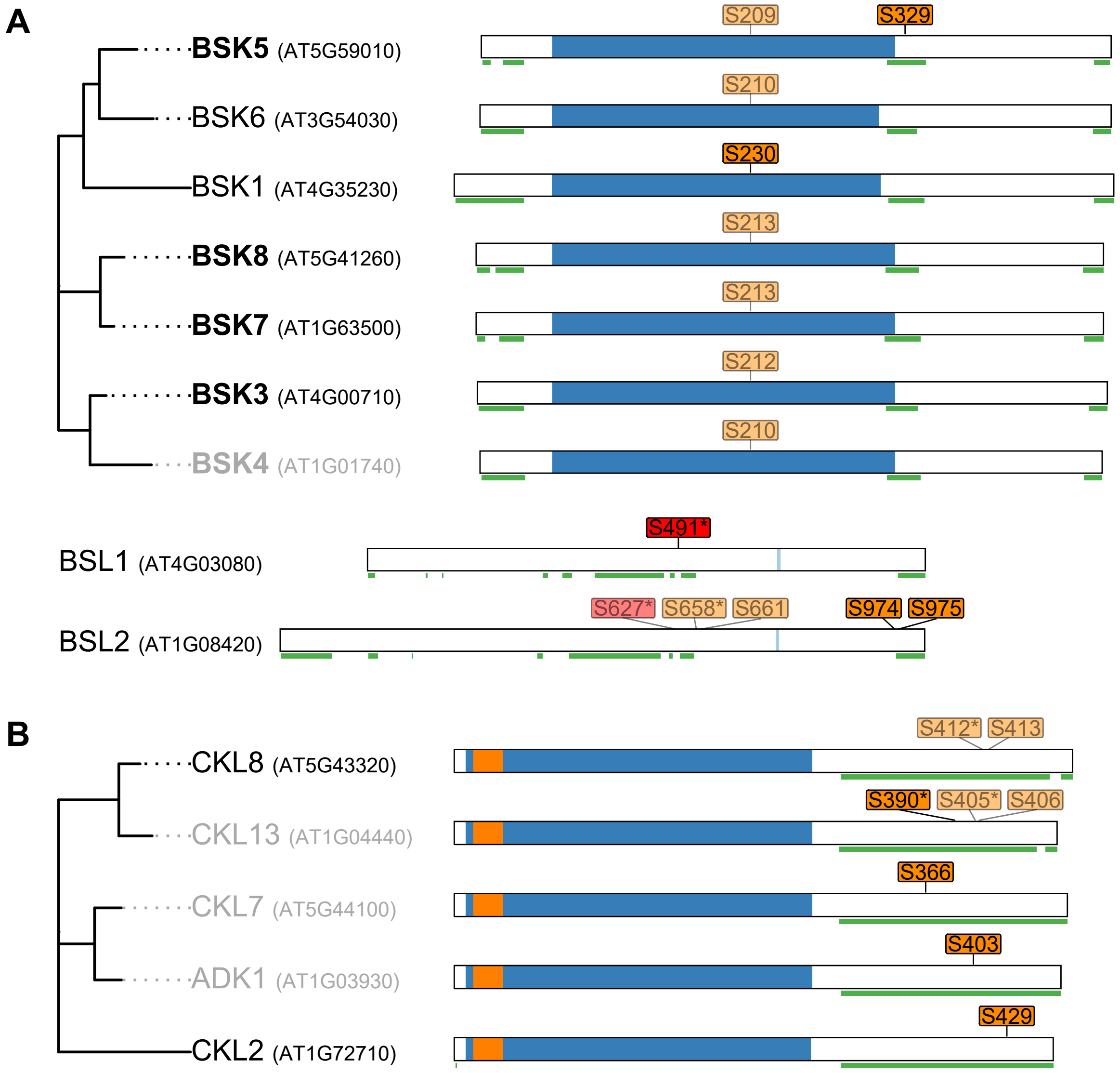

Figure S6. Phosphorylation patterns in BSKs and BSLs **(A)**, and CKLs **(B)**. Gene names in **bold**: high protein abundance; in gray: uncertain presence. Domain architecture is depicted based on ScanProSite and TMHMM predictions: ■KD, ■ATP binding site, ■S/T PP. Disordered regions, based on PrDOS predictions, are underlined in green. pS: detected in both phosphoproteomes, pS: in only one dataset. Non-exclusive phosphopeptides are displayed with transparency. (*) Phosphorylations in an RxxS context.
