# Supplementary material for: OmicIntegrator: A Simple and Versatile Tool for Meta-Analysis
Source: Plants (Basel). 2026 Jan 22;15(2):334. doi: 10.3390/plants15020334 (PMC12845079; doi:10.3390/plants15020334)
Supplement: Supplementary file 1 [file plants-15-00334-s001.zip › Figure S7.pdf]

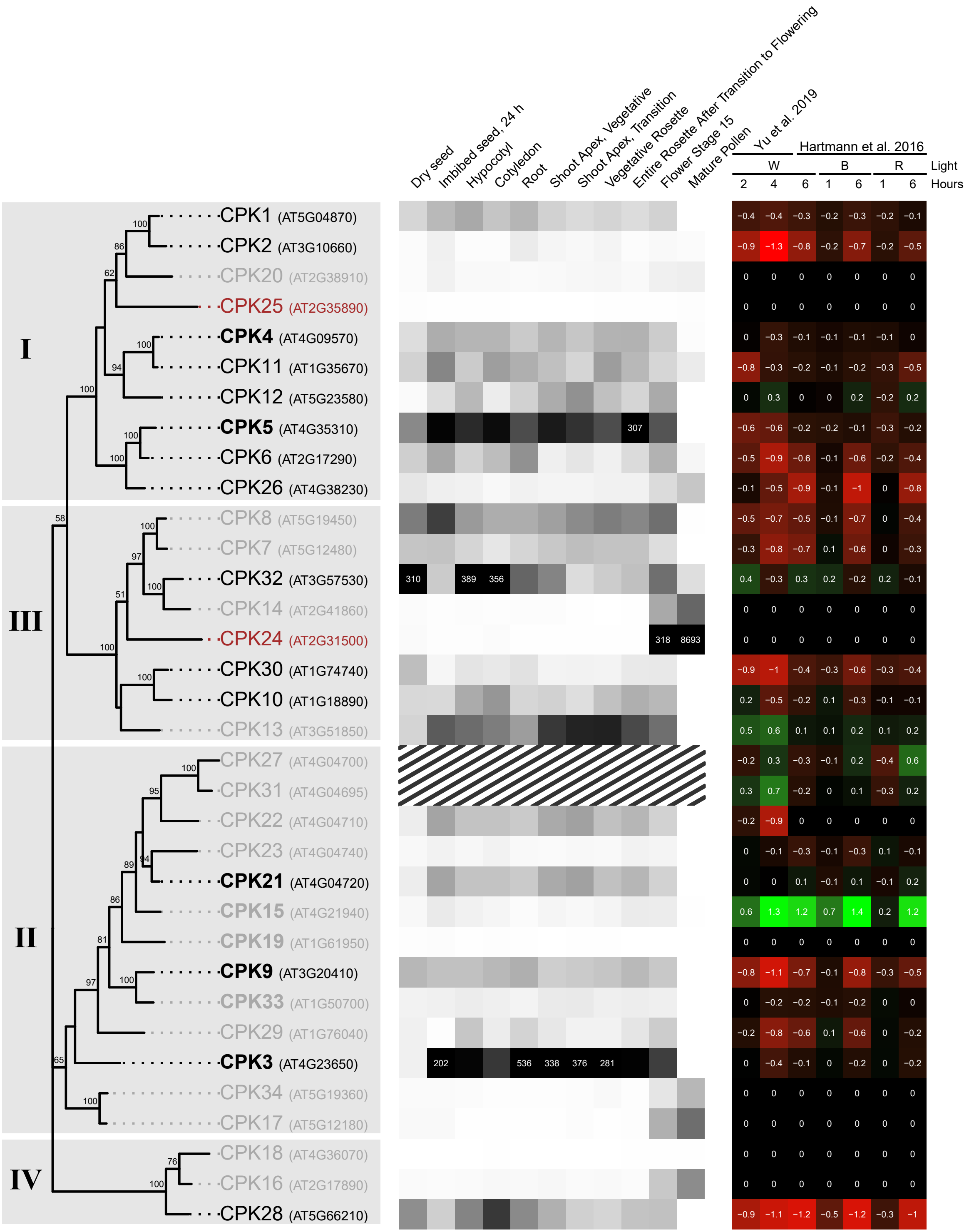

Figure. S7. Tissue-specific expression and light-mediated expression fold of the different members of the AtCPK family. Left panel: CPKs are grouped according to a phylogeny inferred from full-length protein alignments (AGI codes are indicated in brackets). CPKs in **bold**: highly abundant, gray: uncertain presence; brown: absent. Middle panel: heatmap depicting *CPK* tissue expression according to data available at Arabidopsis eFP Browser (Developmental Map dataset). Expression levels of the top-ranked CPK transcripts in each tissue are indicated in numbers inside the black boxes; the gray scale in each tissue is relative to these values. Striped panels in *AtCPK27* and *AtCPK31* indicate that no data is available. Right panel: Fold change [log2(light/dark)] of *AtCPKs* expression in etiolated seedlings exposed to different light treatments for the indicated times. W: white, B: blue, R: red light. Dataset authors are indicated in column headers.
