# Supplementary material for: OmicIntegrator: A Simple and Versatile Tool for Meta-Analysis
Source: Plants (Basel). 2026 Jan 22;15(2):334. doi: 10.3390/plants15020334 (PMC12845079; doi:10.3390/plants15020334)
Supplement: Supplementary file 1 [file plants-15-00334-s001.zip › Figure S8.pdf]

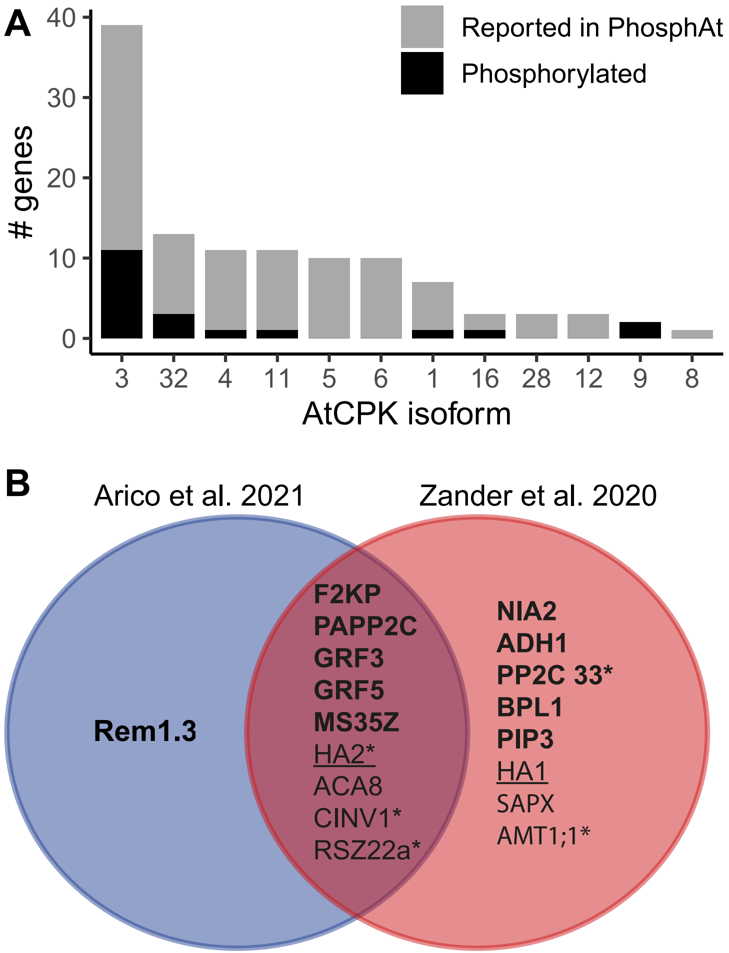

Figure S8. CPK targets reported in the PhosPhAt 4.0 database and detected in the phosphoproteome of etiolated seedlings. (A) Histogram showing the number of experimentally reported CPK targets (PhosPhAt 4.0 DB) corresponding to the CPK isoforms identified in etiolated seedlings. **(B)** Venn diagram of CPK phosphotargets detected in the phosphoproteomes from Arico et al. (2021) and Zander et al. (2020). Targets of CPK3 are shown in **bold**; targets of CPK9 are underlined. (*) indicate phosphoproteins containing a pS within the RxxS consensus motif. ACA8: autoinhibited Ca^{2+}-ATPase isoform 8, ADH1: alcohol dehydrogenase 1, AMT1;1: ammonium transporter 1, BPL1: RNA-binding (RRM/RBD/RNP motifs) family protein, CINV1: cytosolic invertase 1, F2KP: fructose-2,6-bisphosphatase, GRF3/5: general regulatory factors 3/5, HA1/2: H[+]-ATPases 1/2, MS35Z: Ribosomal protein S24/S35, NIA2: nitrate reductase 2, PAPP2C: phytochrome-associated protein phosphatase type 2C, PIP3: plasma membrane intrinsic protein 3, PP2C33: Protein phosphatase 2C family protein, Rem1.3: Remorin family protein, RSZ22a: RNA recognition motif and CCHC-type zinc finger domains containing protein, SAPX: stromal ascorbate peroxidase.
