# Supplementary figures and images for: OmicIntegrator: A Simple and Versatile Tool for Meta-Analysis
Source: Plants (Basel). 2026 Jan 22;15(2):334. doi: 10.3390/plants15020334 (PMC12845079; doi:10.3390/plants15020334)

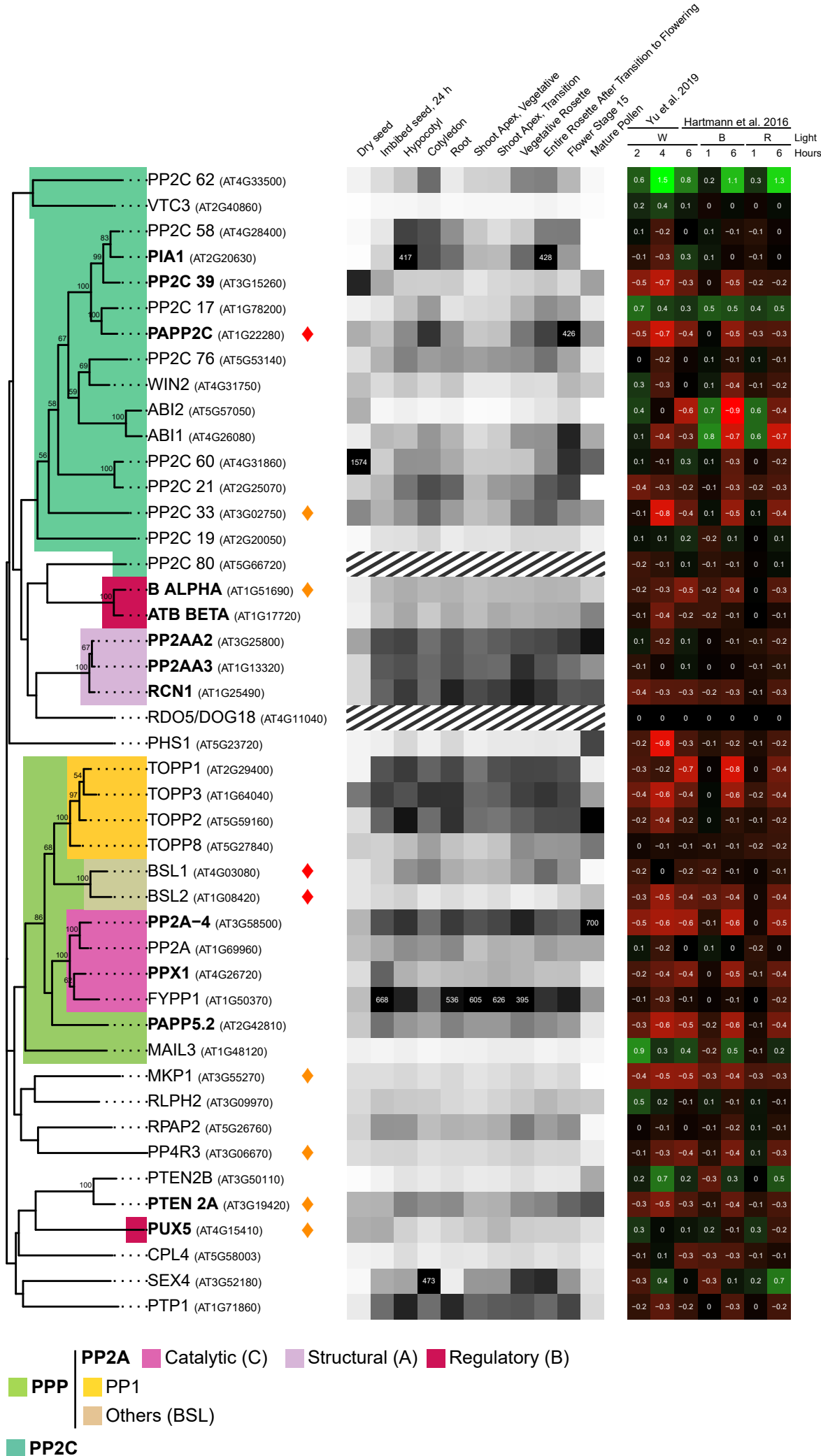

Supplement: Supplementary file 1 [file plants-15-00334-s001.zip › Figure S9.pdf]
