# Supplementary material for: OmicIntegrator: A Simple and Versatile Tool for Meta-Analysis
Source: Plants (Basel). 2026 Jan 22;15(2):334. doi: 10.3390/plants15020334 (PMC12845079; doi:10.3390/plants15020334)
Supplement: Supplementary file 1 [file Figure S5.pdf › Figure S5.pdf]

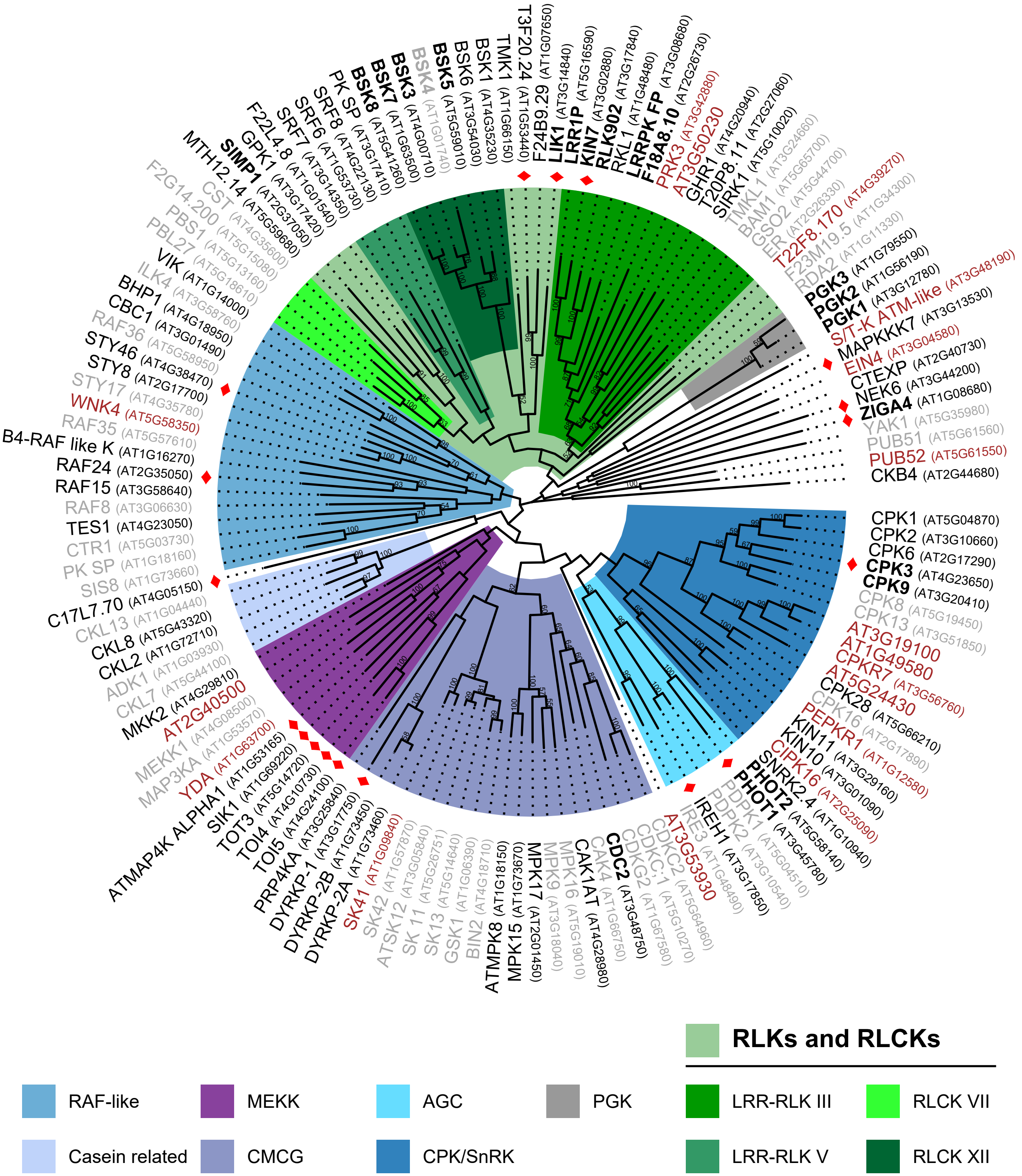

Figure S5. Phylogenetic analysis of 135 phosphorylated PKs detected in etiolated seedlings. The inferred phylogeny was based on full-length protein alignments. Bootstrap support values >50 (from 100 replicates) are shown at the nodes. PK names in **bold**: high protein abundance; in gray: presence not confirmed; in brown: not detected in any proteome. (♦) indicate proteins detected in both phosphoproteomes. Prominent PK families are color-coded; selected RLK subfamilies are indicated.
